# Thermal demands and its interactions with environmental factors account for national-level variation in aggression

**DOI:** 10.3389/fpsyg.2022.911159

**Published:** 2022-09-16

**Authors:** Qingke Guo, Sisi Li, Jinkun Shen, Jianli Lu

**Affiliations:** Faculty of Education, Guangxi Normal University, Guilin, China

**Keywords:** aggressive behavior, moderation effect, natural geographical factors, temperature, thermal demands

## Abstract

Literature shows that psychological phenomena, including values (e.g., individualism vs. collectivism), personality, and behaviors (e.g., prosocial and aggressive behavior), are geographically clustered. The effects of temperature on interpersonal and intergroup aggression have been studied by many social psychologists. To date the interactions between temperature and other geographical factors have not been addressed. This study is aiming to examine the effects of thermal demands and the moderating effects of natural geographical factors on aggressive behavior at national level. Data for 156 societies was obtained from publicly available databases. Consistent with the life-history theory, results of this study showed that aggressive behavior has a positive relationship with heat demands, and a negative relationship with cold demands. Aggressive behavior is also positively correlated with sunlight and altitude, and negatively correlated with coastline vicinity. Forest, coastline vicinity, and rainfall moderated the effect of thermal demands on aggressive behavior. In societies with more forests, with more rainfall, and closer to coastline, the negative effects of cold demands on aggressive behavior are stronger.

## Introduction

Surrounding air temperature or ambient temperature is one of the basic living conditions that influence human social behavior. In recent years the effects of temperature on aggressive behavior have been revealed by many researches (Van Lange et al., 2017; Berman et al., 2020; Potgieter et al., 2022). Aggressive behavior refers to actions that cause physical or psychological harm to others. Broadly speaking, it includes hostile actions causing harm to another person or group, such as bullying, interpersonal conflicts, robbery, political violence, war, and riot (Van Lange et al., 2017). Aggressive behavior sometimes can subjectively be used as a protective mechanism against physical and psychological threats. However, it often objectively impair healthy interpersonal relationships, bring harm and even long-lasting loss (Van Lange et al., 2017). Impulsive personality trait and negative emotionality have been considered as predictors of aggressive behavior (Jones et al., 2011), while good self control abilities and emotion regulation skills can reduce the occurrence of aggression (Roberton et al., 2012). Researchers found that deviant temperature can induce negative emotions, which lead to increased aggression (Van Lange et al., 2017). For example, Rotton and Cohn (2003) found a positive association between temperature and state-level malicious behaviors in the United States. Mares and Moffett (2019) suggested that global warming may enhance aggressive behavior.

Psychological phenomena are spatially clustered (Rentfrow et al., 2013; Steg et al., 2018). Geographical factors (e.g., temperature, rainfall, soil, pathogen prevalence) were found to be associated significantly with many aspects of human psychology, including thoughts, emotions, and behaviors (Hsiang et al., 2013; Rentfrow, 2020). Plenty of studies have found geographical differences in psychological phenomena across nations/regions, such as personality (e.g., Jokela et al., 2015; Stavrova, 2015), cultural values (Van de Vliert, 2013a,b), subjective well-being (e.g., Jokela et al., 2015). These psychological phenomena are closely related to aggressive behavior. To date few studies have examined how environmental factors jointly affect aggressive behavior. This study is aiming to examine the effects of thermal demands and other natural geographical factors as well as their interactions on aggressive behavior at national level. This may throw new light on the understanding of human social psychology (e.g., Van de Vliert and Conway, 2019).

### Cold and heat demands

Human social behaviors can be greatly affected by temperature and other environmental factors, such as relative humidity and the amount of sunlight (Tiihonen et al., 2017). All warm-blooded animals are required to maintain constant levels of body temperature. In humans the relationship between ambient temperature and metabolic rate or body heat production tend to be U-shaped (Van de Vliert, 2013a). Specifically, when ambient temperature is appropriately 22°C, the metabolic rate of human body is minimal. When ambient temperature is lower than this reference point, metabolism increases to produce enough heat (e.g., by shivering) in order to maintain normal temperature (about 37°C) for the human body to survive. When ambient temperature is higher than 22°C, the metabolism also increases to support active cooling (e.g., sweating, panting). And human groups have adapted their behaviors to thermal climate to meet their basic survival needs. Accordingly, the ways people interact with the physical and social environment can be shaped by demanding thermal climate (Van de Vliert, 2013a).

Average temperature has been used as a national level predictor of important psycho-social outcomes (e.g., subjective well-being, freedom). But average temperature is a flawed proxy of demanding thermal climate (Fischer and Van de Vliert, 2011; Van de Vliert, 2013a,b). The main reason is that hotter-than-temperate and colder-than-temperate temperature posed different challenges for humans and consequently showed different effects on human psychological dispositions and behavior (Murray, 2013; Van de Vliert, 2013b; Stevens et al., 2021). For humans 22°C is the most comfortable temperature under which the needs for nutrition and health can be more easily met. Large deviation from 22°C represents less availability of living resources (e.g., animals, plants). Therefore, cross-cultural studies should use variation from the comfortable temperature (both below and above 22°C) instead of average temperature as national level predictor. Van de Vliert (2013b) put forwards the concept of climate demands (i.e., thermal demands) and operationalized it as the sum of the absolute deviations from 22°C across major cities of each country. Considering that coldness and heat may have differential effects on human behaviors, indices of cold demands (to represent survival challenges posed hotter-than-temperate climate) and heat demands (to represent survival challenges posed colder-than-temperate climate) were further proposed (Van de Vliert, 2013b).

### Empirical studies on temperature and aggressive behavior

Abundant research has found the negative impact of high temperature on social behavior and mental health (Berman et al., 2020; Suh et al., 2021). City-center riots, for example, appear to be more common during hot summers and less frequent in cold winters. Cities with higher mean temperature show higher violent crime rates compared with cities with cooler average temperature (Anderson and Anderson, 1996; Anderson et al., 1997). A global level meta-analysis found that high temperature was a major contributor of interpersonal violence and group conflicts (Hsiang et al., 2013). Using the average regional temperature in the United States between 1950 and 1999 as a predictor, Rotton and Cohn (2003) found that annual average temperature was positively associated with physical assault, property crime, and violent crimes. Countries close to the equator experience significantly more aggression and violence than countries far from the equator (Van Lange et al., 2017).

But not all studies found that negative outcomes are caused by hot temperature (Stevens et al., 2021). Russia, Afghanistan, and South Africa are not hot countries, but they all have high domestic violent prime rates (Potgieter et al., 2022). In a global level investigation in 2020,^1^ homicide rate per 100,000 people in South Africa was 33.97, in Russia was 10.82. Using average annual temperature as predictors, Prudkov and Rodina (2019) found that decreased temperature led to increased violent crime in Russia. They further found that in Russia there was more violence in winter and spring months compared with that in autumn months. Using another national level dataset, Iyigun et al. (2017) found that during 1,400 to 1900 low temperature led to an increase in conflict in Europe, North Africa, and the Middle East. The reason is that coldness hampered agricultural production and caused serious social problems such as food shortages and robberies.

Inconsistent results regarding the effects of temperature on aggressive behavior suggest the necessity to take other natural environmental factors into consideration. For example, Sudan and Singapore are hot countries with a yearly average temperature near 28°C, their difference in aggression (Sudan = 1.71, Singapore = −0.38) suggest the influence of natural environmental (e.g., rainfall, forest) and social-economical factor (Van de Vliert and Daan, 2017). These factors and temperature may jointly influence aggression (Hsiang et al., 2013; Abel et al., 2019; Potgieter et al., 2022).

### Environmental factors and aggression

Various aspects of geographic environment can affect human psychological processes and behaviors (Rentfrow et al., 2013). Psychologists have observed personality similarities among people living in the same geographic spaces. This is because physical environments shaped human psychology and behavior. Based on previous literature, we examined the effects of sunlight, forest resource, coastline vicinity, rainfall, as well as altitude on aggressive behavior, and the moderating roles of these environmental variables in the relationship between thermal demands and aggressiveness (Hsiang et al., 2013; Sanciangco et al., 2022). All economic and cultural factors were excluded from this study, because they can be considered as the outcomes of natural environments (Henseler and Schumacher, 2019; Van de Vliert and Conway, 2019).

Sunlight affects people’s psychology and behavior (Esteky et al., 2020). Lambert et al. (2002) found that the human brain production of serotonin was the least in winter, a season short of sunlight. As luminosity increased, serotonin production elevated rapidly. aan het Rot et al. (2008) found that increased natural light intensity led to more positive social interactions and fewer disputes. Therefore, this study hypothesized that societies with more sunlight have fewer aggressive behaviors. But the relationship between sunlight and social behavior may not be linear. Bright light can enhance positive behavior, but can also amplify negative behavior. For example, bright light leads chronically interdependent self-construal individuals to engage in positive behaviors (i.e., more prosocial behaviors), but leads chronically independent self-construal individuals to engage in negative (i.e., less prosocial) behaviors (Esteky et al., 2020).

Forests are familiar environment in human evolutionary history. And human physiological functioning is adapted to this environment (Ikei et al., 2017; Sanciangco et al., 2022). Forests make people more relaxed and comfortable, and help them alleviate negative emotions such as anger-hostility, tension-anxiety, depression, and fatigue. Walking in a forest leads to decreased heart rate and heart rate variability, and lower blood pressure and pulse rate (Song et al., 2019), blood pressure and stress level (Ikei et al., 2017). This suggests that forest environment can reduce the occurrence of aggressive behavior, because negative emotions such as anger, hostility, anxiety, and tension can trigger aggressive behavior (Roberton et al., 2012). This study hypothesized that societies with more forest resource have fewer aggressive behaviors.

Coastline vicinity (as calculated by dividing coastline length by land area), indicating the mean distance from the continent to the ocean, is also an important natural environmental factor influencing human behavior. Coastal and offshore areas have relative milder climates and much wider space for human activities than inland areas. Literature shows that people living in comfortable climates are less likely to experience physiological arousal (Anderson et al., 1997). At the seaside (e.g., sandy beaches), people can easily find open areas to interact with others, thereby satisfying the need for affiliation and establishing beneficial interpersonal relationships (Oishi et al., 2015; Stieger et al., 2022). Compared to coastal environments, mountain areas are not attractive to individuals who are agreeable or extravert (Götz et al., 2020). These individuals are more likely to live in coastal and offshore areas (e.g., Jokela et al., 2015). Previous literature found a stable negative correlation between agreeableness and aggressive behavior, while the relationships between other personality traits and aggressive behavior were inconsistent (Jones et al., 2011; Hosie et al., 2014). Therefore we hypothesized that coastal societies had fewer aggressive behaviors than landlocked societies.

Water is a key indicator of livability. Indeed, precipitation may have played a key role in culture evolution (e.g., Van de Vliert et al., 2018; Van de Vliert and Van Lange, 2019). Severe drought hampered crop growth and resulted in food shortages (Abel et al., 2019). As a result, less rainfall led to persistent violent clashes, especially in underdeveloped societies (Von Uexkull et al., 2016). Therefore, we hypothesized that societies with more rainfall have fewer aggressive behaviors than drier societies.

The effects of high altitude on social behavior tend to be negative, because high altitude can hamper human psychological functioning. At high altitude people are more likely to show behavioral and cognitive disturbances, severe depression and anxiety,and bipolar disorders (e.g., Bahrke and Shukitt-Hale, 1993; Kious et al., 2019). Literature shows that irritable and aggressive moods were very likely to develop at high altitude (Bahrke and Shukitt-Hale, 1993). At high altitude areas, human empathic abilities can be severely damaged. And moral judgment also becomes difficult because humans are costumed to behave in places close to the sea level (Banasiewicz et al., 2014). Therefore, this study hypothesized that there were more aggressive behaviors in high altitude societies compared with in low altitude societies.

These findings suggest that natural environmental factors and temperature can jointly influence aggressive behavior (Van de Vliert and Conway, 2019). Based on existing literature, this study put forward two main hypotheses:

*H1*: Heat demands, cold demands, and natural environmental factors influenced aggressive behavior at national level.

*H2*: Natural environmental factors played moderating roles in the associations between heat and cold demands and aggressive behavior at national level.

## Materials and methods

This study used previously aggregated population-level mean scores that are publicly available. Society (a country or region that has its own government) is the unit of analysis. These scores have been analyzed by other researchers (e.g., Van de Vliert, 2011, 2013a; Van de Vliert and Daan, 2017) and have been proved valid. Cold demands and heat demands were used as two independent variables. Aggressive behavior, as indicated by intergroup conflict, was the dependent variable. Sunlight, forest resource, coastline vicinity, rainfall, and altitude were mainly used as moderators though their main effects on aggression were also the research interest of this study. We first acquired the scores of temperature and aggression for 156 societies from Van de Vliert (2011, 2013a), then collected the population-level scores of other variables for these societies.

### Cold demands and heat demands

Cold demands and heat demands were calculated using mean temperature of major cities in each country, weighted by population. We used the dataset provided by Van de Vliert (2013b).

### Rainfall

Data for average monthly rainfall of each country were obtained from the World Bank Climate Change Knowledge Portal.^2^ The data was first averaged across year (2009 to 2018), then the grand mean of rainfall (mm) of each country were calculated.

### Sunlight and Forest resource

The grand mean of solar radiation (100 kw·h/m^2^) of each country during 1991–2010 was used as an indicator of sunlight. Data were obtained through the weather information inquiry software (Meteonorm version 7) access to the worldwide irradiation data.^3^ Latitude and longitude of each society were determined according to Van de Vliert and Van Lange (2019), and Google Maps. Data for forest resource of each society, as indicated by forest-to-land area ratio, was collected during 2009 to 2016 and averaged across years. The data was provided by the United Nations Food and Agriculture Organization (FAO) on the website of the World Bank.

### Coastline vicinity and altitude

Coastline vicinity was calculated by dividing coastline length by land area of a society. The ratio indicates the average distance from land area to seashore. Data were obtained from the World Factbook published by the Central Intelligence Agency.^4^ Data for altitude of each society was also obtained from the CIA World Factbook. This study used the average altitude (m) of each society as an indicator.

### Aggressive behavior

This study used the intergroup conflict indicator developed by Van de Vliert and Daan (2017) as the measure of national level aggression. This indicator was the average of some standardized national level measures: the index of domestic conflict and violence,^5^ press repression (Van de Vliert, 2011), and business costs due to crime and violence. These measures involved interpersonal aggression and violence, and intergroup conflict, such as political violence, war, and riot.

Literature shows that the world’s climate changes are negligibly for millennia. This warrants the use of different sources of data with slightly inconsistent time span (Van de Vliert et al., 2018). Expectation maximization imputation approach was used to replace a small amount of missing values. Finally, the valid sample included 156 societies. SPSS (Version 26) was used in correlation analysis. The PROCESS macro for SPSS (model 1, Hayes, 2013) was employed to estimate the moderation models using 5,000 bootstrap samples.

## Results

### Correlation among research variables

Correlation among research variables were calculated (Table 1). The results showed that: (1) Cold demands significantly and negatively correlated with aggressive behavior; (2) Heat demands significantly and positively correlated with aggressive behavior; (3) Sunlight significantly and positively correlated with aggressive behavior; (4) Coastline vicinity significantly and negatively correlated with aggressive behavior; (5) Altitude significantly and positively correlated with aggressive behavior; These results suggest that thermal demands and natural environmental factors both are associated with national level aggression.

**Table 1:** Correlations among research variables (*N* = 156).

variable	*M*	*SD*	1	2	3	4	5	6	7
1. Cold demands	37.15	25.95							
2. Heat demands	21.54	7.82	−0.58^***^						
3. Sunlight	17.02	3.88	−0.68^***^	0.57^***^					
4. Forest resource	28.36	21.07	−0.03	−0.26^**^	−0.28^***^				
5. Coastline vicinity	26.16	83.61	−0.12	−0.03	−0.08	−0.12			
6. Rainfall	87.47	59.65	−0.45^***^	−0.10	−0.05	_0.56_^***^	0.14		
7. Altitude	627.93	565.97	0.12	−0.22^**^	0.23^**^	−0.12	−0.20^*^	−0.15	
8. Aggression	0.0026	0.78	−0.34^***^	0.25^**^	0.54^***^	−0.14	−0.25^**^	0.02	0.31^***^

* *p* < 0.05,

** *p* < 0.01, and

*** *p* < 0.001.

### Moderation effects of natural geographical environment

To explore how the joint effects of temperature and environmental factors on aggressive behavior, we used both cold demands and heat demands as independent variables, and five natural environmental variables as moderators. The results were shown in Table 2.

**Table 2:** Moderating effects analysis (*N* = 156).

	Regression equation		Significance of coefficient		Significance of model	
	Outcome	Predictor	*β*	95%*CI*	*R^2^*	*F*
Model 1	Aggression	Constant	0.01	[−0.17, 0.19]		
		Cold demands (CD)	0.07	[−0.12,0.26]		
		Sunlight	0.58^***^	[0.39,0.78]		
		CD× Sunlight	0.02	[−0.16, 0.19]	0.30	21.62^***^
Model 2	Aggression	Constant	−0.01	[−0.15, 0.14]		
		Cold demands (CD)	−0.31^***^	[−0.46, −0.16]		
		Forest resource (FR)	−0.18^*^	[−0.33, −0.04]		
		CD × FR	−0.18^*^	[−0.33, −0.04]	0.17	10.45^***^
Model 3	Aggression	Constant	−0.04	[−0.18, 0.11]		
		Cold demands (CD)	−0.41^***^	[−0.56, −0.26]		
		Coastline vicinity (CV)	−0.48^***^	[−0.70, −0.26]		
		CD × CV	−0.32^*^	[−0.61, −0.03]	0.22	14.65^***^
Model 4	Aggression	Constant	−0.16	[−0.33, 0.01]		
		Cold demands (CD)	−0.53^***^	[−0.71, −0.36]		
		Rainfall	−0.40^***^	[−0.61, −0.19]		
		CD× Rainfall	−0.36^**^	[−0.57, −0.15]	0.20	12.29^***^
Model 5	Aggression	Constant	−0.004	[−0.14, 0.14]		
		Cold demands (CD)	−0.38^***^	[−0.52, −0.23]		
		Altitude	0.34^***^	[0.19, 0.50]		
		CD× Altitude	0.04	[−0.11, 0.18]	0.24	16.09^***^
Model 6	Aggression	Constant	−0.02	[−0.18, 0.14]		
		Heat demands (HD)	−0.10	[−0.29,0.08]		
		Sunlight	0.61^***^	[0.42,0.80]		
		HD× Sunlight	0.03	[−0.13, 0.19]	0.30	21.98^***^
Model 7	Aggression	Constant	0.01	[−0.15, 0.17]		
		Heat demands (HD)	0.24^**^	[0.07, 0.40]		
		Forest resource (FR)	−0.09	[−0.25, 0.07]		
		HD × FR	0.05	[−0.11, 0.21]	0.07	3.86^*^
Model 8	Aggression	Constant	0.004	[−0.15, 0.15]		
		Heat demands (HD)	0.26^**^	[0.10, 0.41]		
		Coastline vicinity (CV)	−0.21^*^	[−0.38, −0.04]		
		HD × CV	0.12	[−0.17, 0.41]	0.13	7.22^***^
Model 9	Aggression	Constant	−0.003	[−0.16, 0.15]		
		Heat demands (HD)	0.25^**^	[0.09, 0.41]		
		Rainfall	0.05	[−0.11, 0.21]		
		HD× Rainfall	−0.03	[−0.19, 0.13]	0.06	3.52^*^
Model 10	Aggression	Constant	−0.007	[−0.16,0.14]		
		Heat demands (HD)	0.32^***^	[0.15,0.49]		
		Altitude	0.37^***^	[0.19,0.55]		
		HD× Altitude	−0.03	[−0.27, 0.21]	0.20	12.91^***^

* *p* <0.05,

** *p* < 0.01, and

*** *p* < 0.001.

**Figure 1:**
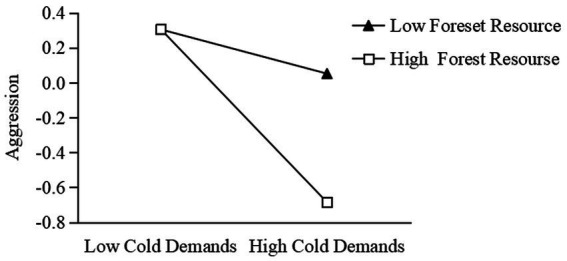
The moderating effect of forest resource in cold demands and aggression.

Model 2 showed that forest resource had a significant moderating effect on the relationship between cold demands and aggressive behavior (the interaction of cold demands and forest resource was significant: *β* = −0.18, *t* = −2.50, *p* = 0.014, 95% confidence interval, CI: [−0.33, −0.04]). Simple slope analysis showed that the negative effect of cold demands on aggressive behavior was significant in the high forest resource condition (high = 1 SD above the centered mean; low = 1 SD below the centered mean; *β* = −0.50, *t* = −5.14, *p* < 0.001, 95% CI: [−0.69, −0.31]); whereas in the low forest resource condition the negative influence of cold demands on aggressive behavior was not significant (*β* = −0.13, *t* = −1.11, *p* = 0.27, 95% CI: [−0.35, 0.10]). See Figure 1 for details. These results suggested that with the increase of forest resource, the negative effect of cold demands on aggressive behavior was enhanced.

**Figure 2:**
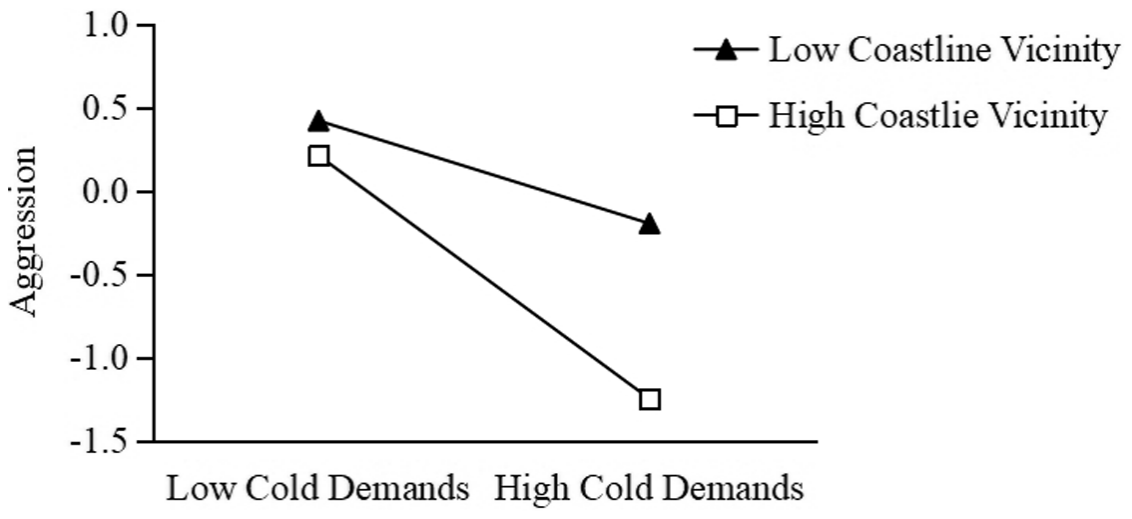
The moderating effect of coastline vicinity in cold demands and aggression.

Model 3 showed that coastline vicinity had a significant moderating effect on the relationship between cold demands and aggressive behavior (the interaction term of cold demands and coastline vicinity was significant: *β* = −0.32, *t* = −2.18, *p* = 0.03, 95% CI: [−0.61, −0.03]). Simple slope analysis showed that the negative effect of cold demands on aggressive behavior was significant in the high coastline vicinity condition (*β* = −0.73, *t* = −4.08, *p* < 0.001, 95% CI: [−1.08, −0.38]). In the low coastline vicinity condition, the negative influence of cold demands on aggressive behavior was still significant (*β* = −0.31, *t* = −3.96, *p* < 0.001, 95% CI: [−0.46, −0.15]), but the regression coefficient was much smaller (Figure 2). These results suggested that in societies near the sea, cold demands can more strongly reduce aggressive behavior.

**Figure 3:**
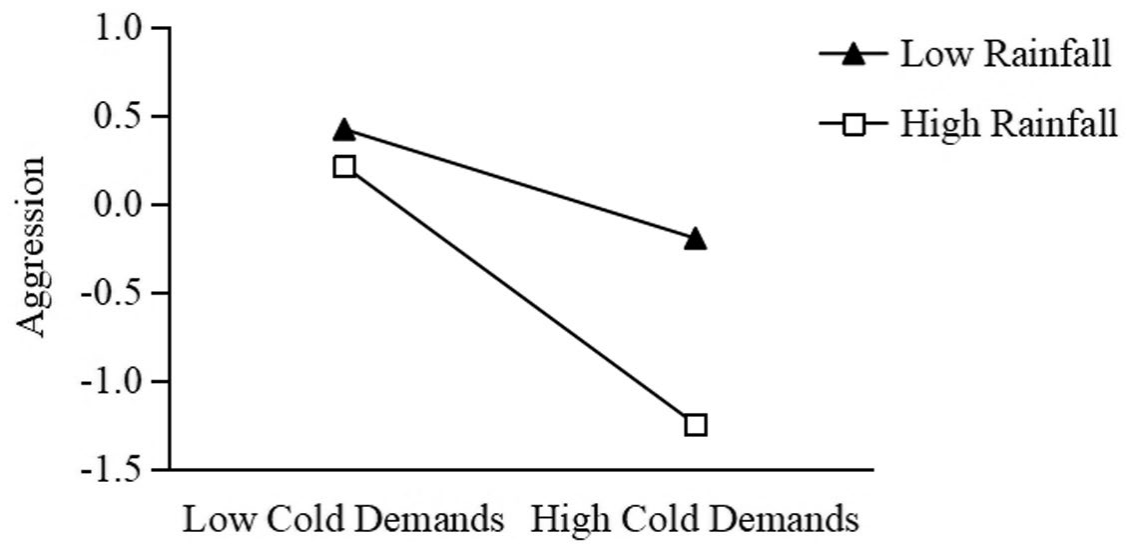
The moderating effect of rainfall between cold demands and aggression.

Model 4 showed that rainfall had a significant moderating effect on the relationship between cold demands and aggressive behavior (the interaction term of cold demands and rainfall was significant: *β* = −0.36, *t* = −3.36, *p* = 0.001, 95% CI: [−0.57, −0.15]). Simple slope analysis showed that the negative effect of cold demands on aggressive behavior was significant in the high rainfall condition (*β* = −0.90, *t* = −5.41, *p* < 0.001, 95% CI: [−1.23, −0.57]). In the low rainfall condition, the negative influence of cold demands on aggressive behavior was not significant (*β* = −0.17, *t* = −1.55, *p* = 0.12, 95% CI: [−0.38, 0.05]), see Figure 3. These results suggested that with the increase of rainfall, the negative effect of cold demands on aggressive behavior was enhanced.

These results suggested that some natural environmental factors played moderating roles in the relation of temperature and aggressive behavior. Surprisingly, heat demands did not show interaction effect with any of the environmental factors.

## Discussion

### Heat demands, cold demands, environmental factors and aggressive behavior

This study is aiming to investigate whether environmental factors influence aggression and the temperature-aggression association, which is substantially fulfilled. Consistent with Hypothesis 1, this study found that heat demands, cold demands, and some natural environmental factors were significantly associated with aggressive behavior at national level. A significant positive correlation between heat demands and aggressive behavior was consistent with previous studies (e.g., Rotton and Cohn, 2003; Larrick et al., 2011; Hsiang et al., 2013). Anderson et al. (1997) posits that situational variables (e.g., high temperature) and individual variables (e.g., genetic characteristics, trait aggression, attitudes towards violence) together affect a person’s aggressiveness. In hot weathers individuals experience high physical arousal, which triggers more aggression. The Routine Activity Theory (Rot and Cohn, 2001) suggests that crime rates are higher in summer because there are more interpersonal interactions during hot months.

The inhibitory effect of coldness on aggressive behavior can be explained by life history theory (Hill, 1993). Lower temperature, and especially larger degrees of seasonal variation in climate, call for individuals and groups to adopt a slower life history strategy: a greater focus on the future (vs. present), and a stronger focus on self-control. These characteristics of individuals are important factors in curbing aggression and violence (Van de Vliert and Daan, 2017). Another explanation for this result is that people interact less frequently at colder (vs. hotter) weather, decreasing the incidence of aggressive behavior (Rotton and Cohn, 2003).

Sunlight was significantly and positively related to aggressive behavior. One possible reason is that the temperature is relatively high in areas with more sunlight (in this study the correlation coefficient of sunlight and cold demands is −0.68; the correlation coefficient of sunlight and heat demands is 0.57, *p*s < 0.001). Another explanation is that sunlight can enhance negative behavior (e.g., Esteky et al., 2020). Psychiatric studies have found that sunlight may interact with serotonin neurotransmission in triggering impulsivity and suicide (e.g., Vyssoki et al., 2012).

Altitude was significantly and positively related to aggressive behavior. This is consistent with findings that irritable and aggressive emotions are more likely to develop at high altitude (Bahrke and Shukitt-Hale, 1993). At high altitude, human empathic ability can be severely damaged, and moral judgment of behavior becomes difficult (Banasiewicz et al., 2014). The negative physiological responses (e.g., Frisancho, 1993) in high altitude suggest that people are more likely to experience negative emotions and behavioral impulsiveness, which are associated with more aggression.

There was a significantly negative correlation between coastline vicinity and aggressive behavior. That is, coastal societies have less aggression than landlocked societies. This reason may be that the coastal regions are better in meeting people’s needs for affiliation (live with others, cooperate, and interact socially with others) and thereby promoting positive interpersonal relationships (Oishi et al., 2015). In other words, coastal environment can reduce the incidence of destructive interpersonal relationships (Jones et al., 2011; Hosie et al., 2014).

However, forest resource and rainfall were not significantly associated with aggressive behavior. This is inconsistent with Hypothesis 1. The possible reason may be that their effects on aggression are more complicated. Although Hypothesis 1 was only partially supported, this study proved that temperature and natural environmental factors indeed influence the national level aggression.

### The moderating effects of environmental factors

Partly consistent with Hypothesis 2, this study found that coastline vicinity, rainfall, and forest resource moderated the relationship between temperature and aggressive behavior. These results deepened the understanding of the relationship between natural environment and aggressive behavior. This lends more support for the effects of geographical factors on human psychology and behavior (Van de Vliert and Van Lange, 2020).

Firstly, forest resource reinforced the negative impact of coldness on aggressive behavior. That is, forest can enhance the negative effect of cold on aggressive behavior. This may be because that the comfort bring by forest (e.g., Ikei et al., 2017; Song et al., 2019) can further reduce disruptive interpersonal interactions. Coldness restrains aggressive behavior by reducing social interactions (Rotton and Cohn, 2003), and forest resource can reinforce this effect.

Secondly, coastline vicinity reinforced the negative impact of coldness on aggressive behavior. That is, in coastal (relative to landlocked) societies the negative impact of coldness on aggressiveness was much stronger. Greater productivity and convenient transportation provided by the ocean contribute to economic prosperity (Rappaport and Sachs, 2003), therefore people are more willing to move to the seaside. Economic development can greatly improve human competence to overcome the survival challenges caused by adverse living conditions (Rappaport and Sachs, 2003). For coastal societies, the year-round variations in temperature are small and the climate is comfortable, these advantages can enhance the negative impact of coldness on aggression (Van de Vliert, 2013a).

Thirdly, rainfall enhanced the negative effect of cold on aggressive behavior. This is consistent with previous findings that rainfall together with other environmental factors can jointly affect conflict and violence (Van de Vliert and Daan, 2017). Rainy and cold weather restrains human interactions, thereby reducing aggressive behavior (Rotton and Cohn, 2003). In humid weathers people are more likely to engage in productive activities than social activities (Lee et al., 2014). However, this study did not find that sunlight and altitude can moderate the relationship between cold demands and aggressive behavior.

Finally, this study did not found moderating effect of natural geographical factors on the relationship between heat demands and aggressive behavior. This might be due to the fact that coldness has been the main threat to human survival for millions of years,while hot climates (i.e., heat demands) indirectly threatened survival through pathogen prevalence (Gurven and Kaplan, 2007; Murray, 2013; Van de Vliert and Conway, 2019). Human ancestors mainly lived in equatorial regions. Until the utility of fire humans began to expand to colder climatic zones. The absence of moderation effects of natural geographical environmental factors may confirm the proposition that the threats posed by pathogen prevalence cannot be alleviated by others factors (e.g., Van de Vliert and Van Lange, 2019).

Findings of this study may have practical implications in reducing the occurrence of dangerous incidents. For example, large scale crowd gathering during hot summer months should be avoided. Large group activities are encouraged to be hold at low altitude or cooler areas, or in coastal and humid areas. In public places measures are encouraged to be adopted to relieve heat and increase humidity artificially to reduce aggression (Von Uexkull and Buhaug, 2021).

### Limitations and future directions

This study examined the main effects of several geographical factors, as well as their moderating effects in the association between temperature and aggression. These findings lend more support for the geographical hypothesis of psychological phenomena, improving the understanding of national level variations in human psychology and behavior. However, limitations of this study have to be addressed. One significant limitation is that we adopted a convenient sampling method. That is, this study only includes countries that can provide aggregated data. Generalizability of our findings is questionable. Furthermore, findings of this study need to be soundly confirmed in strictly controlled laboratory settings (Gelfand and Lun, 2013) in order to determine the causal link between geographical factors and psychological variables.

## Conclusion

Base on national level data this study found that temperature is a significant predictor of aggressive behavior. Cold demands (temperature lower than the reference point) predict less aggression; while heat demands (temperature higher than the reference point) predict more aggression. A society’s sunlight and altitude were significantly and positively related to aggressive behavior; while coastline vicinity was significantly and negatively related to national level aggressive behavior. Forest resource, coastline vicinity and rainfall reinforced the negative impact of cold demands on aggressive behavior. That is, in regions closer to ocean, with more forest resource, and with more rainfall, coldness has stronger weakening effect on aggressive behavior.

## Data availability statement

The original contributions presented in the study are included in the article/supplementary material, further inquiries can be directed to the corresponding authors.

## Author contributions

QG and JS wrote the original manuscript. SL, JS, and JL collected and analyzed the data. QG and SL revised the manuscript. All authors contributed to the article and approved the submitted version.

## Conflict of interest

The authors declare that the research was conducted in the absence of any commercial or financial relationships that could be construed as a potential conflict of interest.

## Publisher’s note

All claims expressed in this article are solely those of the authors and do not necessarily represent those of their affiliated organizations, or those of the publisher, the editors and the reviewers. Any product that may be evaluated in this article, or claim that may be made by its manufacturer, is not guaranteed or endorsed by the publisher.

## Footnotes

^1^ http://worldpopulationreview.com/

^2^ https://climateknowledgeportal.worldbank.org/general-resources

^3^ https://meteonorm.com/

^4^ https://github.com/factbook/country-profiles

^5^ http://www.visionofhumanity.org/#/page/indexes/global-peace-index/2010
